# Implementation of a Health Promotion Practice Using Individually Targeted Lifestyle Interventions in Primary Health Care: Protocol for the “Act in Time” Mixed Methods Process Evaluation Study

**DOI:** 10.2196/37634

**Published:** 2022-08-19

**Authors:** Emma Nilsing Strid, Lars Wallin, Ylva Nilsagård

**Affiliations:** 1 University Health Care Research Center Faculty of Medicine and Health Örebro University Örebro Sweden; 2 Department of Health and Welfare Dalarna University Falun Sweden

**Keywords:** implementation science, facilitation, practice guideline, lifestyle, health promotion, primary health care, health personnel, qualitative research, quality improvement

## Abstract

**Background:**

There is growing evidence that noncommunicable diseases (NCDs) can be attributable to unhealthy lifestyle habits. However, there has been little application of this knowledge in primary health care (PHC).

**Objective:**

This study aims to evaluate the process and outcomes of a multifaceted implementation strategy for a healthy lifestyle-promoting practice in a PHC setting. This practice is based on national guidelines targeting unhealthy lifestyle habits with a potential risk for NCDs.

**Methods:**

A pre-post implementation study design with a control group is used in a PHC setting in central Sweden. The Medical Research Council guidelines for process evaluation of complex interventions will be applied. The implementation process and outcomes will be assessed using a mix of qualitative and quantitative methods. A strategic sample of up to 6 PHC centers will be included as intervention centers, which will receive a 12-month multifaceted implementation strategy. Up to 6 matched PHC centers will serve as controls. Core components in the implementation strategy are external and internal facilitators in line with the integrated-Promoting Action on Research Implementation in Health Services (i-PARIHS) framework and the Astrakan change leadership model. Data will be collected at baseline, during the implementation phase, and 4-6 months after the implementation strategy. Questionnaires will be sent to roughly 500 patients in every PHC center and 200 health care professionals (HCPs) before and after implementation. In addition, purposeful sampling will be used for interviews and focus group discussions with managers, HCPs, patient representatives, and internal and external facilitators. Use of data from medical records and activity logs will be an additional data source.

**Results:**

Recruitment of PHC centers began in March 2021 and ended in Spring 2022. Based on the planned timeline with the 12-month implementation strategy and 4-6-month follow-up, we expect to collect the final data in Summer 2023.

**Conclusions:**

This study will explain implementation process and outcomes using a multifaceted implementation strategy for a healthy lifestyle-promoting practice in a real-world PHC context. The study is expected to provide new knowledge about the role of facilitators and their contribution to implementation outcomes. These findings can guide policy makers, managers, and PHC staff to integrate health promotion and disease prevention in PHC and provide methodological support to facilitators.

**Trial Registration:**

ClinicalTrials.gov NCT04799860; https://clinicaltrials.gov/ct2/show/NCT04799860

**International Registered Report Identifier (IRRID):**

DERR1-10.2196/37634

## Introduction

### Lifestyle Habits

The leading cause of prolonged disability and premature death worldwide are noncommunicable diseases (NCDs), such as cardiovascular diseases, cancer, chronic respiratory diseases, and diabetes [1-3]. NCDs account for almost two-thirds of deaths globally [2]. This global chronic disease burden is attributed to population demographics (eg, aging, health disparities, and certain risk factors) [3,4]. The major risk factors for NCDs are tobacco use, harmful use of alcohol, low physical activity, and poor nutrition [1,5]. These behavioral risk factors often occur in a cluster, creating a synergetic effect that increases the risk [6]. Individuals with only one of these unhealthy habits die on average 6 years earlier than their counterparts [7]. Recently, the effect of lifestyle interventions targeting these risk factors was shown to be half to almost equal to that of pharmacological treatment for cardiovascular risk factors [8]. Research has shown the importance of adopting a healthy lifestyle through health care interventions [9-11]. Health care systems are encouraged to use prevention and early detection services and improve population health [3], also among children and adolescents [4]. Primary health care (PHC) is a natural arena for this health promotion. As proposed by the World Health Organization, a proactive PHC approach has a crucial role in promoting healthier lifestyles through public contact. PHC professionals should “make every contact count”; that is, making healthy living a priority [12]. Swedish PHC has roughly 40 million visits each year in a population of about 10 million individuals. This large number of visits is an opportunity to promote proactive lifestyle habits. Behavioral and highly modifiable risk factors are well known, and clinical practice guidelines regarding their management have been established [13,14]. Despite the available evidence of the association between risk factors and NCDs, applying this knowledge in a preventive PHC still represents a huge challenge for PHC professionals [15-19].

In 2011 (updated 2018), national clinical practice guidelines for health promotion and disease prevention were published in Sweden, targeting the following unhealthy lifestyle habits: tobacco use, harmful use of alcohol, low physical activity, and poor nutrition [13]. However, the uptake and use of the Swedish guidelines have been low [20]. These guidelines should be considered a complex intervention [21]. They include several lifestyle habits, target managers and diverse health care professionals (HCPs), require expertise and skills in behavior change techniques, and demand a change in routine practice. These Swedish guidelines will be implemented in this study. The guidelines include the following: encouraging patients to fill in a screening form for health behaviors, invite patients with unhealthy lifestyle habits to visits, and discuss and provide individually targeted lifestyle advice, follow-up, and documentation in the patients’ medical record. Further investigations into the implementation process and the professionals’ uptake and use of these guidelines are warranted if we hope to ultimately improve health promotion in the prevention of NCDs.

### Implementation Strategies

Implementation science studies methods to promote the integration of research, such as translating clinical guidelines into routine practice [22]. A theoretical springboard is desirable to understand and explain how and why implementation succeeds or fails, identifies influencing factors, and develops strategies to implement clinical interventions successfully [22]. Many theoretical implementation models and frameworks have been published in recent decades. Today, there is a call to study how such frameworks contribute to more effective implementation [23].

Implementation of clinical interventions (eg, guideline recommendations) implies a change in clinical practice, where the context in which the change takes place, plays an important role [17,24-26]. Implementation strategies, defined as techniques used to enhance the adoption, implementation, and sustainability of a clinical intervention, constitute the “how-to” component of changing health care practice [27]. Common implementation strategies targeting professional behavior change are educational meetings, audit and feedback, printed educational material, local opinion leaders, and tailored implementation strategies [28-30]. The complexity of implementation strategies can vary widely, from a single (discrete) strategy to a combination of strategies creating a multifaceted strategy [27,31,32]. Some authors suggest that implementation strategies should be chosen and tailored to address the context of a given change effort. However, there is little guidance on how to conduct such strategies [30,33]. In a systematic review of methods for designing interventions to change HCPs’ behavior, approaches that identify and prioritize barriers and link strategies to overcome them were proposed, using theory and engaging end users [34]. Moreover, changes in clinical practice were found to be more likely to occur when the HCPs initiated the changes themselves, when the changes featured their active input, and when the changes were seen as well founded and properly communicated [35].

An increasingly used implementation strategy is facilitation, defined as “a process of interactive problem solving and support that occurs in a context of a recognized need for improvement and a supportive interpersonal relationship” [31]. The authors of the integrated-Promoting Action on Research Implementation in Health Services (i-PARIHS) framework describe facilitation as the active ingredient of implementation, implying a dynamic and deliberate process to support implementation [36]. The facilitator role involves assessing and responding to the characteristics of the clinical intervention and the recipients of the intervention in their work context. Facilitators can be external or internal to where implementation occurs, and a combination of the two is frequently reported [37]. They can operate at different organizational levels, from a clinical team to a higher level of the health system. The key is that they (ie, the facilitators) meet the requirements of the role, in terms of their personal attributes, knowledge, and skills [38,39]. Facilitation is judged as a promising strategy [38-41], but there is a need for further research on the role of facilitators and their contribution to implementation outcomes.

This study will follow the recommendation of using frameworks to guide the implementation [42]. The Consolidated Framework for Implementation Research (CFIR) [26] will be used to explore and analyze determinants for the implementation of a healthy lifestyle-promoting practice in PHC based on the Swedish national guidelines for health promotion and disease prevention [13]. Core components in the implementation strategy are internal and external facilitation [36] and steps in a change leadership model [43,44]. The implementation strategies will be prioritized and adapted through discussions with the involved stakeholders [42]. The Medical Research Council (MRC) guidelines for process evaluations of complex interventions will be used in this study for evaluation of the implementation process [45].

### Aims

The overall aim of the Act in Time study is to evaluate the process and outcomes of an implementation strategy for a healthy lifestyle-promoting practice using individually targeted lifestyle interventions in a PHC setting. The specific aims are as follows: (1) to explore what managers, internal facilitators, HCPs, and patient representatives experience as barriers and facilitators when using individually targeted lifestyle interventions in routine health promotion practice; (2) to evaluate the outcomes achieved from implementing the new routine health-promoting practice using medical record data and stakeholders’ perspectives (patients, HCPs, internal and external facilitators, and PHC managers); and (3) to explore the implementation process and the mechanisms of impact.

## Methods

### Design

**Figure 1:**
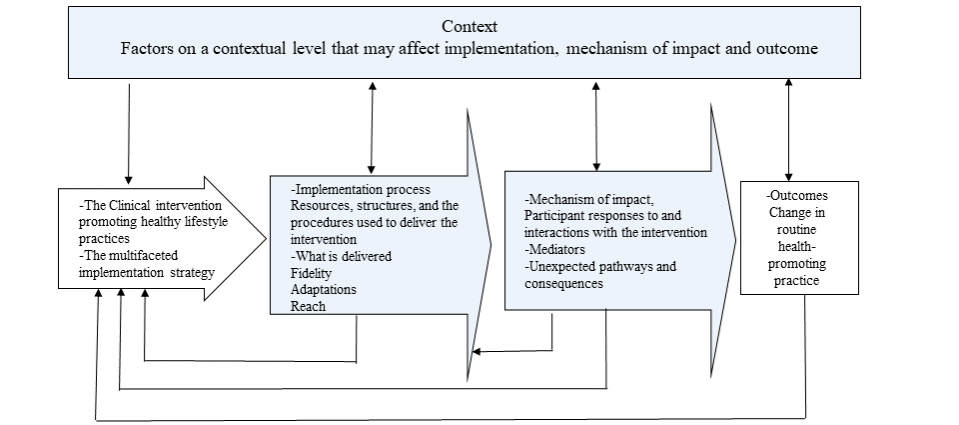
Critical functions of process evaluation and their relations (blue boxes are key components of a process evaluation). Adapted from Moore et al [45] and modified for process evaluation of the Act in Time study.

This study incorporates a pre-post implementation design with a control group [46]. The MRC guidelines for process evaluations of complex interventions are used [45]. The implementation process and outcomes (ie, a change in routine health-promoting practice) will be evaluated using a combination of qualitative and quantitative methods as described in the MRC guidelines. The guidelines highlight the relationships among implementation, mechanisms of impact, and context (Figure 1). All 3 key components need to be evaluated to understand how change is created [45]. This study follows the Standards for Reporting Implementation Studies (StaRI) [47,48].

### Setting and Recruitment

We will conduct the study in a PHC setting in Region Örebro County. The region, located in central Sweden, has 300,000 inhabitants. Region Örebro County has 30 PHC centers, of which 26 are publicly financed and 4 have entrepreneurship contracts with the region. During the project’s initiation in March 2021, the research team informed all PHC managers about the project by email and a presentation at a manager meeting. PHC centers showing their interest in the study are provided with more information. We will use a strategic sampling strategy to achieve variation among PHC centers with respect to size, socioeconomic status, and geographic and rural/urban location. A sample of PHC centers will be contacted by a second invitation email. The first centers willing to participate will be included as intervention centers. Up to 6 centers will be included and up to 6 other centers matched by the factors mentioned above (size, socioeconomic status, and geographic and rural/urban location), will serve as control centers. The control centers will not be offered implementation support. All HCPs employed at the PHC intervention centers will be briefed by their managers and the research group about the study. HCPs (general practitioners, physiotherapists, psychologists, social workers, district nurses, registered nurses, and assistant nurses) having patient visits will be invited to participate in anonymous web surveys. A purposeful sample procedure will be applied for interviews and focus group discussions (FGDs) with HCPs. The theoretical framework (CFIR) will guide the qualitative data collection and analysis [26]. The CFIR provides a structure for approaching complex, interacting, and multilevel implementation processes. The CFIR consists of 5 major domains: intervention characteristics, outer setting, inner setting, characteristics of the individuals involved, and the implementation process. Each domain contains several constructs [26,49]. The interview guides will be based on the constructs of the CFIR [26,50] and focus on the HCPs’ perceptions of barriers and opportunities to integrate a healthy lifestyle-promoting practice at their PHC centers. A modified person-centered process mapping will describe the patients’ perspective on the health promotion practice [51]. A purposeful sample of approximately 8 patients will be recruited from the PHC centers for individual interviews. This interview guide will cover several steps: the process of prevention, contact, investigation, decisions on action/treatment, and follow-up. In each of these steps, the questions will target the patient’s needs and experiences [51]. The research team will discuss the general findings from the interviews and FGDs with the external facilitators and adapt the implementation activities in accordance with these findings. The qualitative data will also be analyzed and results presented in a scientific paper.

### The Clinical Intervention: a Healthy Lifestyle-Promoting Practice

In this study, managers and HCPs will be supported in implementing a healthy lifestyle–promoting practice. In the new routine, HCPs will be expected to encourage patients to fill in a screening form for health behaviors, invite patients with unhealthy lifestyle habits to visits, and discuss and provide individually targeted lifestyle advice, provide follow-ups, and document this in the patients’ medical record. This routine health promotion practice is based on the Swedish national guideline for health promotion and disease prevention, targeting unhealthy lifestyle habits: tobacco use, harmful use of alcohol, low physical activity, and poor nutrition [13]. Before the visit, patients with planned visits to a PHC will be encouraged to fill in a screening form in health care guide 1177 (Swedish: Vårdguiden 1177), a national hub for information and services within health care in Sweden. When unhealthy habits are reported in the screening form, both the patient and the HCP can be prepared and have an opportunity to discuss the information. According to evidence-based recommendations [13], measures should be offered to those with one or more unhealthy lifestyle habits. The recommendations should be recorded in the patient’s medical record using the Swedish classification of health intervention codes. The classification codes are divided into qualified advice, advice, and simple advice for lifestyle habits [13]. Prescribed physical activity and completed screening forms should also be recorded in the patients’ medical record.

### The Multifaceted Implementation Strategy

#### Overview

The implementation strategy is aimed to support the implementation of a health promotion practice at the PHC intervention centers. To accomplish this goal, we will follow a systematic and theory-based approach in 2 steps to select and tailor strategies in accordance with contextual conditions and the HCPs’ needs [26,49]. The multifaceted strategy to support the implementation is based on previous research [30,31] and includes the involvement of target groups, information and interactive educational activities, use of internal and external facilitators, audit and feedback, dialogue, and networking. A change leadership model will also be used to achieve sustained organizational and individual behavior change [43,44].

The implementation strategy will take place over a 12-month period. In the strategy, the managers’ responsibility will be emphasized and an organization will be built to support PHC centers in the application of all steps of the change leadership model [43,44]. External and internal facilitators are central components in the implementation strategy, in line with the i-PARIHS framework [36,37]. In addition, the HCPs’ motivation to participate in the change toward a health promotion practice will be facilitated by focusing on mastery/competence, autonomy, and relatedness. These 3 psychological needs are important for intrinsic motivation [52]. The 4 factors that enhance intrinsic motivation for change will be considered: (1) understanding the purpose for change, (2) opportunity to impact the change, (3) competence to perform the new behavior, and (4) feeling of belonging. The strategies will be supported by structures, including organizational and hands-on tools, quick reference guides, or functional IT systems [44]. An overview of the implementation strategy including actions, actors, target groups, etc, as recommended by Proctor et al [27], is provided in Table 1.

**Table 1:** Specification of Act in Time implementation strategies.

Implementation strategy	External facilitator (EF)	Internal facilitator (IF)	Mandate change	Audit and feedback
Actor	Four organizational developers Competence in quality improvement, implementation, and process management Trained in the change leadership model Experience in working with evidence-based guidelines, knowledge support, and health promotion	2 health care professionals (HCPs) at each primary health care (PHC) center Interest in health promotion and disease prevention Acknowledged as a trustworthy coworker Given priority in the region’s lifestyle education	PHC managers Advisory board members (mangers at the highest management levels in the PHCs’ region)	External facilitators
Actions	Provide context-specific implementation support following the steps outlined in the model of leading change: insight, analyses, planning, and implementing Support the clinical intervention Arrange meetings with managers and IFs at the PHC centers to discuss roles, work structure, strategies, and steps leading to change Distribute educational materials Support change process and provide IFs access to structures, templates, etc Act as a soundboard for IFs Offer IFs individual support and guidance in supporting change, quality improvement, lifestyle habits, and motivational interviewing Offer network opportunities to managers and IFs for peer-learning and reflections Document activities in an activity log	Learning through interacting with EFs Support the implementation of change at their respective PHC center together with the manager, in close collaboration with EFs Support and guide colleagues in change, lifestyle habits, and motivational interviewing Document activities and organizational changes in an activity log	PHC managers mainly responsible for the implementation at their center The advisory board supports managers at the PHC intervention centers and participates in manager networks offered by EFs	Provide feedback based on data from each PHC center’s medical records on the classification of health intervention codes, prescribed physical activity, and numbers of filled screening forms Communicate with IFs and managers and facilitate their reflections on areas in which they have performed well and areas that can be improved to reinforce their health-promotion practice in accordance with their goals
Action target	IFs and managers at the PHC centers	HCPs/colleagues at their PHC center	Managers and HCPs at the PHC centers	IFs and managers at the PHC centers
Temporality	Twelve-month support, more intense in the beginning and decreasing over time	Act as IF during the 12-month implementation period	Planning phase and during the 12-month implementation period	Feedback will be provided monthly and before meetings with IFs during the implementation period
Dose	Based on the needs of IFs and managers, about 1 meeting a week (2 hours) at each PHC center IF Network every second month (2 hours) Manager Network every third month (2 hours)	Implementation dedicated as a work assignment, approximately 10% of working hours	Continuous manager support Participate in Manager Network every third month (2 hours)	Feedback will be provided monthly and before meetings with IFs
Implementation outcomes affected	Change in routine health-promoting practice and sustainability	Change in routine health-promoting practice and sustainability	Feasibility and acceptability	Change in routine health-promoting practice and sustainability
Justification	Facilitation [36-38,53,54] Activity log [55]	Facilitation [38] Activity log [55]	Change leadership model [43,44]	Audit and feedback [56]

#### External Facilitators

Four organizational developers at the development unit in the Örebro region are contracted as external facilitators to provide context-specific implementation support at the PHC intervention centers. During spring 2021, they were provided further education by a certified change leader in accordance with the Astrakan change leadership model [44]. In this advanced education, the change leadership model was discussed on the basis of the characteristics and challenges in the proposed research project. The external facilitators (n=4) work in pairs to support the internal facilitators and managers at the PHC centers. They will work systematically with the PHC centers to accomplish a shared vision, intrinsic motivation, adequate competence, resources to support the change, and an anchored plan for change [53]. The experiences of external facilitators of the implementation and their thoughts from meetings with managers and internal facilitators will be discussed bimonthly by members of the research group (YN and ENS). The discussions at these meetings enable a context-specific and interactive process concerning planning, coordination, learning, and tailoring implementation strategies for the specific PHC intervention centers at the micro level. The sessions are important because of the fluid nature of facilitation over time, aiming to develop the external facilitators’ skills and improve the implementation of the intervention [39,53]. The external facilitators will regularly document their performed activities, intent, duration, and individuals involved, as well as perceived barriers and facilitating factors in an activity log [55].

#### Internal Facilitators

Managers at each PHC intervention center will appoint 2 HCPs as internal facilitators. They will receive support from the external facilitators [54] regarding the clinical intervention and in supporting each step of the change process. Every week, they will report all performed activities and organizational changes in an activity log related to the implementation at their respective PHC intervention centers [55].

#### Mandate Change

According to the change leadership model [43,44], the managers at the PHC intervention centers need to accept responsibility for change as they are accountable for the transition into a more health promotion practice at their centers. An advisory board anchors the project at the highest management levels in the PHCs’ region.

#### Audit and Feedback

Data from each PHC center’s medical records will be used in communication with managers and internal facilitators at the PHC intervention centers.

### Data Collection

**Figure 2:**
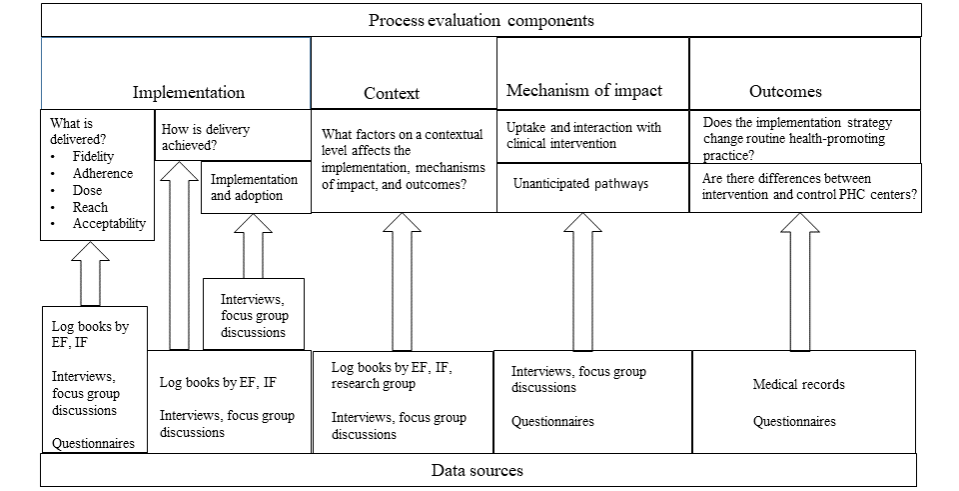
Overview of the Act in Time study’s process evaluation components, research questions, and data sources. EF: external facilitator; IF: internal facilitator; PHC: primary health care. Adapted from Saarijärvi et al [58].

**Figure 3:**
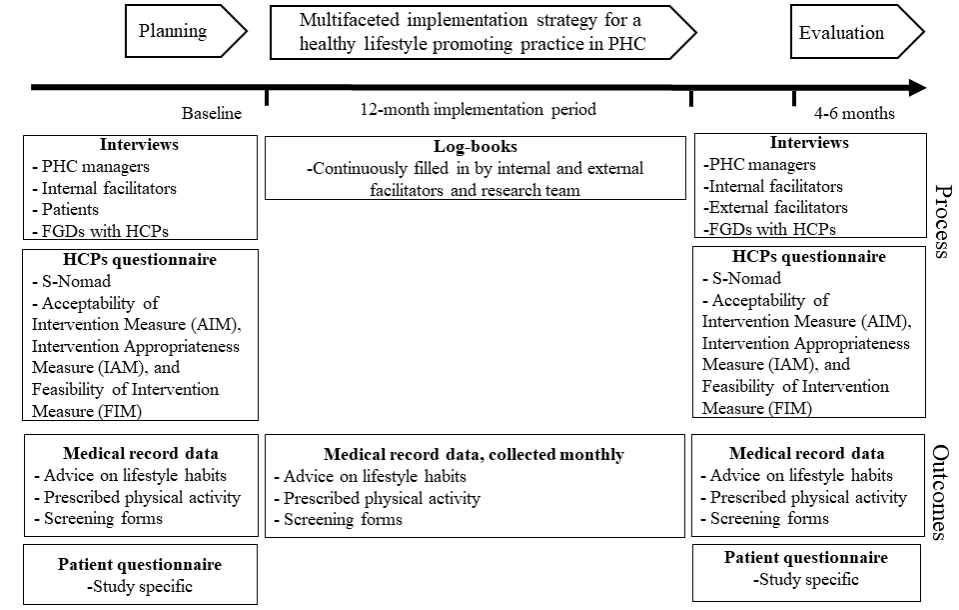
An overview of data collection including the timeline, target group, and methodology. FGD: focus group discussion; HCP: health care professional; PHC: primary health care.

For rigorous process evaluations, data will be collected before, during, and after accomplishing the implementation strategy [45,57]. An overview of the data collection process is provided in Figure 2, and a more detailed overview including the timeline is provided in Figure 3.

#### Implementation Outcome Evaluation

To evaluate changes in HCP health promotion routine, data will be collected from both intervention and control PHC centers as follows:

1. Registered medical record data concerning the frequency of individually targeted lifestyle interventions by HCPs, as shown in the administrative registration of clinical interventions including simple or qualified lifestyle advice and prescriptions for physical activity. Data will be collected monthly.
2. Registered medical record data for screening forms on lifestyle habits will be completed by patients before visiting the PHC center. Data will be collected monthly.
3. A subset of patients visiting a PHC center for approximately 1 week will receive a questionnaire through text message (SMS) or post asking whether they had been encouraged to fill in the screening form before their visit. They will be asked whether lifestyle habits were discussed and whether advice was given. The survey will be provided in 8 languages to reach those who did not speak Swedish. The survey will be distributed to up to 3000 patients at baseline and follow-up (approximately 500 patients per PHC center). Finally, 2 reminders will be sent to all participating patients.

With regard to the aforementioned data collected (points 1-3), we will only retrieve medical records data from patients aged ≥18 years.

#### Implementation Process Evaluation

The following will occur at the PHC intervention centers:

1. We will query HCPs whether they perceive the health promotion practice as acceptable, appropriate, and feasible, using a validated and short questionnaire [59,60] distributed by email with a link to the questionnaire. Two reminders will be sent. Data will be collected from approximately 200 HCPs.
2. We will evaluate how HCPs have integrated the health promotion practice to their daily work using the validated questionnaire S-Nomad [61-63]. This survey will be distributed with the short questionnaire described above (in point 1).
3. We will perform individual interviews with PHC managers (n=10-12) and internal facilitators (n=10-12) and FGDs with HCPs on capturing their experiences of the implementation process. Up to 6 FGDs will be moderated with a purposeful sample of 5-7 HCPs at each intervention center.
4. We will ask internal and external facilitators to write structured activity logs during the implementation strategy to assess the fidelity, dose, and reach of the implementation intervention.
5. We will conduct individual interviews with the 4 external facilitators once the implementation strategy is completed at all PHC intervention centers. An independent researcher—not involved in the study design, the implementation strategy, or its evaluation—will conduct these interviews.

With regard to the aforementioned points 1-3, we will collect data approximately 1 week before starting and 4-6 months after ending the implementation strategy.

### Data Analysis

Separate teams will analyze outcome and process evaluations [45].

#### Quantitative Data

Quantitative data will be analyzed using SPSS Statistics for Windows (version 27.0, IBM Corp). Data from patients’ questionnaires and medical records and HCPs’ questionnaires will be analyzed using generalized linear mixed models [64]. Specifically, Poisson and logistic models will be used for count and proportion outcomes, respectively. Parameters will be estimated using maximum likelihood estimation. Fixed effects will include intervention, month, and sex, whereas random effects will include nested patients at PHC intervention centers. Model checking will involve the inspection of residual and autocorrelation plots. To quantify the magnitude and direction of intervention effects, we will use the incidence rate ratio (IRR) from the Poisson model and odds ratio (OR) from the logistic model. To determine statistical significance, a *P* value of <.05 will be considered. CIs will be adjusted for simultaneous inference where several contrasts are presented.

#### Qualitative Data

All interviews and FGDs will be digitally audio recorded and transcribed verbatim. The transcribed texts will be imported into NVivo (version 12; QSR International) to manage and code data. Qualitative content analysis will be used to analyze the data from the interviews and FGDs [65]. A deductive approach will be used for the data collected at baseline, where the constructs in the implementation framework CFIR will guide the analysis [49,50]. An inductive analysis will be used to describe the HCPs’ and managers’ perceptions of the health promotion practice concerning the CFIR constructs. Information from process mapping with patients and data from activity logs will be compiled and processed qualitatively and quantitatively. The process and outcome evaluation data will be integrated to determine whether implementing the health promotion practice succeeded [45,66].

### Ethical Considerations

The Act in Time study was approved by the Swedish Ethical Review Authority (DNRs 2020-06956, 2021-00912 and 2021-05825-02). Participation is voluntary, all data will be handled confidentially, and only authorized personnel will have access to the data. The managers, HCPs, and internal and external facilitators participating in the interviews and FGDs will be prompted to provide written informed consent. The audio files and transcripts will be coded and saved on a password-protected server. The code key and other relevant material will be stored in a safe locker. The data gathered from medical records will be anonymized and protected. We will check the lists of patients visiting the PHC intervention centers and not send questionnaires to deceased individuals. Patients and PHC staff will fill out questionnaires anonymously. The study will comply with the tenets of the Helsinki declaration [67] and the recommendation in the StaRI checklist [47,48]. The COREQ (COnsolidated criteria for REporting Qualitative research) checklist will be used when reporting findings from the qualitative studies [68].

## Results

Recruitment of PHC centers began in March 2021 and ended in Spring 2022. As of June 2022, five PHC centers have been recruited and baseline data collection is completed. Based on the planned timeline with the 12-month implementation strategy and up to 4-6–month follow-up, we expect to collect data until Summer 2023. Data collected from interviews and FGDs at baseline will be analyzed during spring 2022, and these results are expected to be published in Autumn 2022. Results from the evaluation of process and outcomes of the implementation strategy are expected to be published between 2024 and 2025.

## Discussion

### Expected Findings

The results of the Act in Time study can help to better understand the implementation process and what may be effective (or ineffective) in influencing HCPs and organizational change toward a more proactive and health promotion practice in PHC. Insight into potential paths to improvement to inform broader implementation strategies and scale up implementation may also be derived from our study [23,69].

Strong evidence has been found showing a link between behavioral risk factors and NCDs [1,5-7]. National health promotion and disease prevention guidelines have been established to combat these risk factors [13,14]. Nevertheless, applying these recommendations is still a challenge for PHC professionals [15-19]. In a systematic review Wändell et al [15] identified several barriers and facilitators that PHC professionals face in preventing NCDs. Lack of time, reimbursement, education, and counseling skills were the most common obstacles. Positive attitudes toward prevention and awareness of the effectiveness of health checks were the most commonly reported facilitators [15]. This study will identify barriers and facilitators and adapt the implementation strategy in accordance with these determinants, the local context, and the stakeholders’ needs, as previously proposed [30-32,34]. The engagement of stakeholder groups [34,35] (ie, managers and HCPs) is considered crucial throughout the implementation strategy and may enable change in HCPs’ behavior [34]. The study is supported by regional political and higher manager levels, which, together with creating an advisory board, will strengthen the opportunities to implement the project. The role of leadership has previously been acknowledged as necessary in the implementation and sustainment of evidence-based practice in health care [70,71]. It is crucial to have acceptance and support from the leadership at different levels [42]. Facilitation is a core component of the implementation strategy. External facilitators will reinforce the strategy and support internal facilitators and managers in changing practice [38-40]. This study will contribute to important knowledge on the role of external and internal facilitators in changing the routine health promotion practice.

### Strengths and Limitations

The proposed study has several strengths. A novelty of this study is the combination of implementation science [23,26] and a change leadership model [43,44]. Such an approach seeks to achieve tangible and sustained individual and organizational change. Moreover, we will use medical record data as feedback to the PHC intervention centers during the implementation period. The medical record data serve as a formative evaluation of the implementation strategy aiming to adapt and strengthen it. In addition, the discussions among external facilitators and the research group might enable beneficial adjustment of the implementation strategy [42]. This kind of refinement of the implementation strategy in response to accumulated data, operating as an adaptive and variable response to context, is considered a desirable feature of implementation research [66].

We will perform a comprehensive evaluation of the process and outcomes of a multifaceted implementation strategy for implementing a healthy lifestyle–promoting practice in a PHC setting using qualitative and quantitative methods in accordance with MRC guidelines [45]. We will use a pre-post design, which helps examine the impact of a complex implementation strategy in a real-world setting when a randomized controlled trial is not suitable or when assessing the adoption and adherence to guideline recommendations by HCPs [46,72]. However, the design may mean less control over confounding factors. Instead of power calculation, we apply a strategic sampling method to achieve maximum variation in PHC centers and thus strengthen the external validity and generalization of our findings. The external validity is dependent on the study design but also the social, economic, political, and organizational context in which the study is performed, which must be considered when translating the findings into a different context. Data will be collected before, during, and after implementation, allowing a more rigorous assessment of the implementation process [57]. A strength is the use of validated questionnaires and registered medical record data. The separate process and evaluation teams are other study strengths [45].

This study faces challenges due to the ongoing COVID-19 pandemic and organizational changes in the region. These challenges have led to staff shortages and a heavy workload, which may affect the ability of PHC centers to participate in the study and for HCPs to answer questionnaires. We will send questionnaires to approximately 500 patients per PHC center on 2 occasions. However, we expect a relatively low response rate as patients may be reluctant to click on the link to the questionnaire in the text message. Thus, reminders will also be sent by post.

### Conclusions

This study will contribute to the implementation processes and outcomes of a multifaceted implementation strategy for a health promotion practice in a real-life PHC setting. More specifically, we expect to enhance knowledge and understanding of the role and contribution of internal and external facilitators for implementation outcomes when changing the routine practice and contributing to the development of facilitators’ training and role in future work. Our findings could help to guide policy makers, managers, and HCPs in integrating health promotion and disease prevention into PHC practice.

## Abbreviations

CFIR: Consolidated Framework for Implementation Research
COREQ: COnsolidated criteria for REporting Qualitative research
FGD: focus group discussion
HCP: health care professional
i-PARIHS: integrated-Promoting Action on Research Implementation in Health Services MRC, the Medical Research Council
MRC: Medical Research Council
NCD: noncommunicable disease
PHC: primary health care
StaRI: Standards for Reporting Implementation Studies

## Data Availability

The data sets generated and analyzed during the current study are not publicly available because they contained information that could compromise the integrity of research participants but are available from the corresponding author on reasonable request.
